# Sea-blue histiocytosis: an unusual small-bowel endoscopic finding

**DOI:** 10.1055/a-2559-4147

**Published:** 2025-03-27

**Authors:** Adriana Vaz Safatle-Ribeiro, Isabella Nicacio de Freitas, Ricardo Artigiane Neto, Marcela Paes Rosado Terra, Ana Carolina Campos, Klaus Mönkemüller

**Affiliations:** 1 Department of Gastroenterology, Hospital das Clínicas, Faculdade de Medicina da Universidade de São Paulo, São Paulo, Brazil; 2 Department of Pathology, Universidade Federal de São Paulo, São Paulo, Brazil; 3 Department of Gastroenterology, Virginia Tech Carilion School of Medicine, Roanoke, United States

A 67-year-old man, diagnosed with heart failure and atrial fibrillation, was referred for anterograde double-balloon enteroscopy to investigate iron deficiency anemia requiring blood transfusion and duodenal polyposis diagnosed on upper digestive endoscopy. Colonoscopy was normal. The patient also had chronic liver disease, with small esophageal varices, hepatosplenomegaly, and thrombocytopenia. Laboratory examination showed hemoglobin of 8.6 g/dL, alkaline phosphatase 207 IU/L, and gamma-glutamyl transferase 83 IU/L.

Double-balloon enteroscopy revealed numerous sessile, elevated, pedunculated, and multilobulated lesions, some forming bridges into the lumen, distributed throughout the duodenum and the entire portion of the examined proximal and mid jejunum ( Fig. 1 , Video 1 ).

**Fig. 1:**
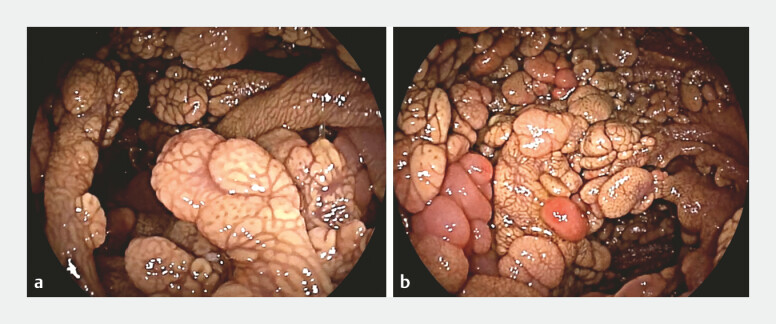
Double-balloon enteroscopy images. **a** Sessile and pedunculated lesions in the duodenum. **b** Multiple polypoid lesions in the jejunum.

Double-balloon endoscopy showed duodenal and jejunal involvement of sea-blue histiocytosis.Video 1

Histology of duodenal and jejunal biopsy samples demonstrated subepithelial foamy macrophages with cytoplasmic granularity on hematoxylin and eosin staining ( Fig. 2 **a** ). Due to the accumulation of mucosubstances including glycolipids in the cytoplasm, the histiocytes appeared with lipopigment granules that were stained by periodic acid–Schiff ( Fig. 2 **b** ) and by Ziehl–Neelsen stain ( Fig. 2 **c** ). No bacteria were observed.

**Fig. 2:**
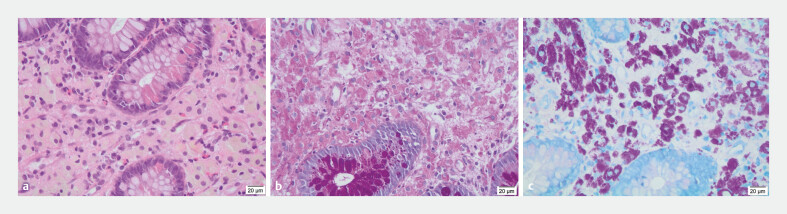
Histology images. **a** Hematoxylin and eosin staining demonstrated numerous macrophages. **b** Periodic acid–Schiff staining showed macrophages with cytoplasmic granularity. **c** Ziehl–Neelsen staining revealed lipopigment granules.

Sea-blue histiocytosis has already been described in several clinical conditions such as lipid storage diseases (Niemann–Pick), thalassemia, myelodysplastic and myeloproliferative syndromes, liver diseases, and in patients on parenteral nutrition 1 2 . Sea-blue histiocytosis is a very rare lysosomal lipid storage disease that can be found in various organs such as bone marrow, liver, spleen, and small intestine 3 . Possible differential diagnoses are Gaucher disease and Whipple disease 2 . Thrombocytopenia, anemia, splenomegaly, and hepatomegaly may be present, and a few cases may progress to liver cirrhosis and portal hypertension 4 5 .

Endoscopy_UCTN_Code_CCL_1AC_2AH

## Notes

Endoscopy E-Videos https://eref.thieme.de/e-videos
*E-Videos* is an open access online section of the journal *Endoscopy* , reporting on interesting cases and new techniques in gastroenterological endoscopy. All papers include a high-quality video and are published with a Creative Commons CC-BY license. Endoscopy E-Videos qualify for HINARI discounts and waivers and eligibility is automatically checked during the submission process. We grant 100% waivers to articles whose corresponding authors are based in Group A countries and 50% waivers to those who are based in Group B countries as classified by Research4Life (see: https://www.research4life.org/access/eligibility/ ). This section has its own submission website at https://mc.manuscriptcentral.com/e-videos .
